# Beyond Microbiological Analysis: The Essential Role of Risk Assessment in Travel-Associated Legionnaires’ Disease Outbreak Investigations

**DOI:** 10.3390/pathogens14101059

**Published:** 2025-10-20

**Authors:** Antonios Papadakis, Eleftherios Koufakis, Vasileios Nakoulas, Leonidas Kourentis, Theodore Manouras, Areti Kokkinomagoula, Artemis Ntoula, Maria Malliarou, Kyriazis Gerakoudis, Katerina Tsilipounidaki, Dimosthenis Chochlakis, Anna Psaroulaki

**Affiliations:** 1 Department of Clinical Microbiology and Microbial Pathogenesis, School of Medicine, University of Crete, 71110 Heraklion, Greece; kokareti@gmail.com (A.K.); artemisntoula@gmail.com (A.N.); maria.malliarou.ger@gmail.com (M.M.); surreydimos@hotmail.com (D.C.); 2 Public Health Authority of the Region of Crete, 71201 Heraklion, Greece; 3 Civil Protection of the Region of Crete, 71201 Heraklion, Greece; elkoufakis@crete.gov.gr; 4 Department of Materials Science and Engineering, University of Crete, 70013 Heraklion, Greece; tman@iesl.forth.gr; 5 Department of Hygiene and Epidemiology, Faculty of Medicine, University of Thessaly, 41222 Larissa, Greece; nakoulasb@gmail.com (V.N.); leokourentis@uth.gr (L.K.); 6 Regional Laboratory of Public Health of Crete, School of Medicine, 70013 Heraklion, Greece; k.gerakoudis@eody.gov.gr (K.G.); tsilipoukat@gmail.com (K.T.)

**Keywords:** *L. pneumophila*, environmental surveillance, Legionnaires’ disease, outbreak investigation, risk assessment, Water Safety Plan (WSP)

## Abstract

Between April and May 2025, an outbreak of travel-associated Legionnaires’ disease (TALD) occurred, involving six cases at a hotel in Crete, Greece. Including two cases reported in 2023 and two additional cases from 2016 to 2017, ten cases were associated with this accommodation site. All TALD cases were reported by the European Legionnaires’ Disease Surveillance Network (ELDSNet). In compliance with the European Centre for Disease Prevention and Control (ECDC) surveillance and investigation protocols for hotels associated with the patient’s stay, local public health authorities conducted on-site inspections at the hotel by collecting water samples and performing risk assessments, while simultaneously recording the required epidemiological, environmental, and physicochemical data. A total of 181 statistically analyzed water samples showed positive rates for *L. pneumophila* of 12.71% (95% CI: 7.86–17.56) for (≥50 CFU/L) and 6.08% (95% CI: 2.60–9.56) for (≥1000 CFU/L). Risk assessments identified 18 stagnation points, systemic maintenance deficiencies, and high cumulative structural (30/52) and water (36/71) system risk scores. Low microbiological positivity of water samples does not necessarily equate to low risk, thus necessitating continuous risk assessment, implementation of Water Safety Plans (WSPs), and integrated monitoring by accommodation facilities to prevent LD cases.

## 1. Introduction

Travel-associated Legionnaires’ disease (TALD) is defined as a clinical case of Legionnaires’ disease (LD) associated with travel and exposure to contaminated water systems in accommodation facilities such as hotels, resorts, and cruise ships [1,2,3,4,5,6,7]. In particular, according to the European Legionnaires’ Disease Surveillance Network (ELDSNet), the TALD case definition is “each case of a traveler who stayed at or visited a commercial accommodation site 2–10 days before clinical onset and has not been associated with other cases of LD in the last two years.” A cluster is identified when two or more travelers who have stayed at or visited the same commercial accommodation site within 2 to 10 days prior to the onset of illness and experience the onset of illness within the same two-year period. Similarly, according to European Union procedures, if “two or more travelers have stayed at the same accommodation within 14 days before the onset of symptoms”, this is considered relevant even if the illnesses are reported in Member States other than where the accommodation is located [8,9,10,11].

An outbreak is characterized by the occurrence of two or more cases of illness that are closely connected in time, typically within weeks rather than months, and in location. This is often accompanied by suspicion or evidence of a shared source of infection, which may or may not be supported by microbiological data, such as a common location indicated by travel history [2].

Travelers may encounter illness either during their journey or upon returning home, particularly when the travel duration exceeds one week. This is due to the incubation period, which ranges from 2 to 10 days, with an average of 5 to 6 days [12]. During their travels, individuals may stay at multiple hotels across different cities or even countries according to their itinerary [8,9,10,11,13]. While a single case at a hotel does not confirm it as the infection source, multiple cases linked to the same accommodation within a short period suggest it as the likely source, warranting immediate investigation [2,14,15,16]. For rapid clusters, clinicians must test for LD symptoms such as myalgia, fatigue, cephalalgia, non-productive cough, and pyrexia, especially in patients who have recently traveled to affected areas [17,18].

Worldwide, most cases of LD are caused by *L. pneumophila*, most commonly serogroup 1 (SG1), due to its ability to form biofilms, resist water disinfection, and survive within protozoa and human macrophages. These characteristics contribute to its persistence in artificial water systems, resulting in severe pneumonia, particularly in immunocompromised individuals and older adults. [19,20,21,22]. Given that confirmed cases of LD are mainly associated with *L. pneumophila*, environmental surveillance protocols are designed to detect these species, resulting in under-detection of cases. A comprehensive approach to the environmental investigation of all *Legionella* species facilitates the early detection of potential risks, as non-*pneumophila* species, while less frequently associated with clinical disease, pose a threat under specific environmental or host conditions. Pontiac fever, a mild, non-pneumonic, flu-like form of legionellosis, is caused by *Legionella* bacteria but not exclusively by *L. pneumophila* [23,24].

The complexity of water distribution systems, characterized by intermittent flow and factors that contribute to water stagnation, such as variable water demand, aging infrastructure and piping, blind or dead legs, and insufficient residual disinfectants, constitutes the primary, though not exclusive, risk factors for the proliferation of *Legionella* [1,25,26]. Beyond the most common sources such as cooling towers and hot water systems, *Legionella* infections may also be linked to cold water systems and recreational areas (including pools and pool showers), water features, and hotel gardens [2,25,27,28].

Hotels directors advised to maintain cold water temperatures under 20 °C, hot water temperatures above 50 °C, and free chlorine concentrations between 0.2 mg/L and 5 mg/L to effectively reduce *Legionella* colonization, it is advised [29,30].

The chemical composition of the water intended for human consumption is of significant importance. Hard water facilitates the formation of deposits that protect biofilms. In contrast, moderate water softening can reduce residual disinfectant levels, thus promoting the proliferation of opportunistic bacteria such as *Legionella* spp. and *Pseudomonas aeruginosa* [31,32,33,34].

Local public health authorities must monitor TALD cases by implementing simultaneous inspection, water sampling, and comprehensive risk assessment. This strategy is essential for identifying and managing the links between accommodation facilities and confirmed cases, clusters, or potential outbreaks. Recent studies have demonstrated that clusters associated with a particular hotel often reflect a larger community outbreak with extensive consequences [35,36,37,38].

A prior study utilizing surveillance data from Crete, Greece (2020–2025), identified persistent colonization of *L. pneumophila*, particularly SG1, which was detected in 38.59% of hotel water systems. Notably, hotels exhibited high positivity rates in 2025, despite the implementation of preventive measures [39,40,41]. Studies conducted in the post-COVID era at accommodation sites have revealed that following the initial three years of district-wide implementation of health protocols, there has been a relaxation in hotel hygiene standards. This has resulted in significant non-compliance with the mandated preventive measures and an increase in positivity rates [28,42,43,44].

A review conducted in 2024 emphasized that the design of plumbing systems, water chemistry, and devices used for aerosol production significantly affect the persistence and risk of *Legionella* transmission [42]. Several recent outbreaks, including those associated with hot tubs and cruise ships, underscore the continuing importance of *Legionella* control in tourist environments [25].

The current study aimed to investigate the underlying cause of the recurring TALD cases in a tourist facility in Crete (2016–2018 and 2023–2025), evaluating the microbiological, physicochemical and structural risk factors. This was deemed necessary given that despite the long period since the first cases, the cluster of cases in 2024 and 2025 was again associated with it. Thus, it was thoroughly studied whether the current surveillance limits and preventive measures in the water supply system of the tourist accommodation were capable of adequately preventing these accumulations.

## 2. Materials and Methods

### 2.1. Study Setting and Period

This case study concerns a three-star hotel in a small seaside municipality on Crete’s Island. The hotel operates seasonally from May to October, with a capacity of 94 rooms and 181 beds for accommodating tourists mainly through travel agencies. The facility was built in 1982, with a radical renovation around 2005.

Based on the on-site inspection and the review of the architectural plans, the building’s layout was examined, including the basement, ground floor, four upper floors designated for guest accommodation, and an outdoor recreational area.

Detailed characteristics of the hotel water networks are summarized in Table 1. The network is made of PVC pipes, with three boilers of 2000 L each connected in series. Hot water is circulated throughout the building using a circulation pump and a collector system. Water heating is provided by a gas boiler, assisted by solar panels. Seasonal operation runs from May to October.

Fire protection relied on hose reels directly supplied from the potable water network, without a dedicated firefighting system, while climate control was provided by autonomous air conditioning units. The recreational water facilities comprised an outdoor swimming pool, supplied with municipal water, disinfected manually with sodium hypochlorite, and continuously recirculated through a sand filter, as well as two decorative fountains with recirculated but non-disinfected water. Additionally, a distinct reverse osmosis network, incorporating chlorine dioxide dosing, supplied post-mix machines, kitchen appliances, and ice makers. In conclusion, irrigation was implemented through a drip system that utilized water from the primary supply. Moreover, the primary disinfectant agent used for the water treatment was sodium hypochlorite, supplemented by hydrogen peroxide. In 2025, hydrogen peroxide was replaced by chlorine dioxide as the secondary disinfectant component.

Eighteen points with potential for water stagnation were identified within the hotel’s water distribution system, comprising five dead ends (27.8%) and 13 stagnant lines (72.2%). Backflow prevention devices were installed and found to be in compliance with NF EN 1717.

The environmental investigation and risk assessment period covers the timeline from 2016 to 2018, 2023 and 2025 and involves only the years in which TALD cases have been reported. During May 2025, extensive sampling was performed before the decision to close the facility was made by the local Public Health authority and following the implementation of control measures for the reopening of the facility.

### 2.2. Case Definition and Diagnostic Criteria

A confirmed case of LD was defined as a clinical or radiological diagnosis of pneumonia with laboratory confirmation of *L. pneumophila* infection, consistent with international case definitions [45,46,47].

In this study, confirmed cases, as reported by ELDSNet, were identified by urinary antigen detection of the *L. pneumophila* SG1. These criteria are in accordance with the Council of State and Territorial Epidemiologists (CSTE) 2020 definition for legionellosis surveillance and are fully aligned with the case definitions adopted by the ECDC, the World Health Organization (WHO), and the ELDSNet for TALD [45,48,49,50].

To allocate the epidemiological characteristics of LD cases in Crete in 2025 and to contextualize the cluster observed in the hotel under investigation, all confirmed cases reported from January to September were presented. TALD cases were confirmed through urinary antigen detection of *L. pneumophila* serogroup 1 (SG1) or by PCR detection of *Legionella* DNA in respiratory specimens. Environmental monitoring targeted all *Legionella* species in accordance with national and international surveillance guidelines. Data on confirmed LD cases reported across Crete from April to September 2025 were included solely to provide epidemiological context and were not incorporated into the subsequent risk assessment or environmental investigation focused on the hotel under study.

### 2.3. Sample Collection

A sampling plan was developed based on the architectural design of the hotel, encompassing both cold and hot water distribution networks, as well as recreational water facilities. The plan included storage tanks, boilers, recirculation lines, risers, outlets (such as showers and taps), and outdoor recreational water facilities. A total of 205 environmental samples were collected, comprising 174 bulk water samples and seven swab samples for the enumeration of *Legionella* spp. In addition, 16 targeted water samples were collected beyond the routine sampling scheme, specifically aimed at exploring potential associations between microbiological findings (e.g., *L. pneumophila*, *P. aeruginosa* and indicator bacteria) and the effectiveness of water disinfection practices. Finally, eight additional water samples were collected for analysis of chemical parameters.

Microbiological sampling was performed according to the relevant ISO protocols implemented throughout the period 2016–2017 and 2023–2025, which included the selection of sampling points, rinsing procedures where required, and aseptic handling.

Water samples were collected for microbial analysis using sterile 1 L bottles containing 20 mg/L sodium thiosulfate to neutralize the residual disinfectant. The samples were transported to the Regional Public Health Laboratory of the Crete Region and the Regional Public Health Laboratory of the Thessalia Region in insulated refrigerators at 5 ± 3 °C and were processed within 24 h of collection.

The temperature (°C), residual free chlorine (mg/L), and pH values were measured on-site at the time of sampling using calibrated portable instruments. The compliance limits for the physicochemical parameters were based on the accepted guidelines: hot water ≥ 50 °C, cold water ≤ 20 °C and free chlorine ≥ 0.2 mg/L.

### 2.4. Microbiological and Chemical Analysis

The detection and quantification of *Legionella* spp. in water samples followed ISO 11731-2 [51,52]. Water samples were concentrated by membrane filtration and resuspended in 10 mL of sterile Ringer’s solution (1:40 dilution). Aliquots (200 μL) were inoculated onto Buffered Charcoal Yeast Extract (BCYE) agar, and Glycine Vancomycin Polymyxin Cycloheximide (GVPC) agar. Inoculations were performed: (a) directly after filtration, (b) after heat treatment at 50 °C for 30 min, and (c) after acid treatment (0.2 mol/L HCl, pH 2.2). The detection limit was 50 CFU/L. The plates were incubated at 36 ± 2 °C with increased humidity for up to 10 days. Suspected colonies were subcultured to confirm *Legionella* growth with confirmed *L. pneumophila* isolates serotyped to distinguish (SG)1 from SG2–15. To distinguish between serotypes 2 and 15 within the SG2-15 cluster, capsular serotyping was performed using latex agglutination with type-specific antisera. Indicator bacteria (Enterobacteriaceae) were analyzed using ISO 9308-1:2014 [53], Total Viable Count (TVC)@22 °C ISO 6222:1999 [54], coliform bacteria ISO 9308-1:2014 [53], Amd1:2016, *E. coli* ISO 9308-1:2014 [53] & Amd1:2016, and intestinal enterococci ISO 7899-2:2000 [55].

Physicochemical parameters were determined according to the Standard Methods for the Examination of Water and Wastewater (APHA, 24th edition) and were validated in-house using HACH-based protocols. The parameters analyzed included calcium, magnesium, nitrates, nitrites, ammonium, sulfates, chlorides, pH, conductivity, total hardness, total alkalinity, and turbidity [56]. Physicochemical measurements were also performed in situ, including temperature (°C) after 2 min of flow, free residual chlorine (mg/L) using DPD colorimetry, and pH and conductivity using multiparameter probes.

#### Swab Samples

Swab samples were collected from faucets and shower outlets according to ISO 19458:2006 [57]. Sterile cotton swabs with sodium thiosulfate (20 mg/L) were inserted into the outlet surfaces, including aerators and showerheads. The swabs were placed in sterile tubes containing 10 mL of sterile water with sodium thiosulfate, stored at 2–8 °C, and processed within 24 h. Each swab was vortexed in the transport fluid, and the suspension was concentrated and examined for *Legionella* spp., following ISO 11731:2017 [51,52] procedures, alongside water samples.

### 2.5. DNA Extraction and Whole Genome Sequencing

Selected *L. pneumophila* isolates were forwarded for DNA extraction using the ExtractMe Genomic DNA Kit (Blirt S.A., Gdańsk, Poland), following the manufacturer’s instructions. One nanogram of each extract was used for library preparation with the Nextera XT DNA Library Preparation Kit (Illumina, San Diego, CA, USA) according to the manufacturer’s recommendations. The library size distribution was assessed with a QIAxcel Advanced capillary electrophoresis system (Qiagen, Hilden, Germany) equipped with a 12-capillary QIAxcel DNA high-resolution cartridge, and DNA concentration was quantified using the Qubit dsDNA HS Assay Kit (Invitrogen, Thermo Fisher Scientific, Wilmington, DE, USA) on a Qubit 5 fluorometer. Whole-genome sequencing (WGS) was performed on a NextSeq 2000 System (Illumina) using a NextSeq 1000/2000 P1 Reagent Kit (300 cycles) to produce 2 × 150 bp paired-end reads.

### 2.6. Whole Genome Sequencing Data Analysis

Raw sequencing reads were assessed for quality using FastQC and filtered with fastp to remove low-quality bases and adapter sequences [58,59]. Filtered reads were assembled de novo using SPAdes v3.15.0 with default parameters and the “--careful” option enabled to minimize assembly errors [60]. In silico sequence-based typing was performed with legsta. To generate the cgMLST profiles, the chewBBACA software (v3.4.4) was used to apply the cgMLST scheme defined by Moran-Gilad et al. (2015), which includes 1521 core genes [61]. Allelic distances were calculated as the sum of differing loci among all core genes in the generated cgMLST schema. The resulting allelic profiles were analyzed with GrapeTree to construct Minimum Spanning Trees (MSTs) for each *L. pneumophila* isolate.

### 2.7. Risk Assessment (RA)

A combined risk assessment (RA) framework was applied to integrate international and national tools for *Legionella* control. The methodology incorporated the CDC Water Management Toolkit for developing a Water Management Program (WMP), the European Technical Guidelines 2017: minimizing the risk of *Legionella* infections in building water systems, and the national Greek NPHA checklist supported by structured scoring systems for building and water system characteristics [62].

RA was performed on-site at the investigated facility to systematically evaluate the potential for *Legionella* proliferation and exposure. The process involved a step-by-step inspection and mapping of the entire water distribution system, with the identification of hazardous conditions (e.g., stagnation, suboptimal temperatures, inadequate disinfection, and external hazards) and corresponding control measures.

Overall, RA followed a multi-tool approach, integrating three standardized and internationally recognized checklists and scoring systems to ensure a comprehensive evaluation of the system design, operation, maintenance, and verification practices.

#### 2.7.1. ECDC Facility Inspection Tool for TALD [63]

This tool was applied to assess six main domains: (i) competence and training of personnel; (ii) domestic cold and hot water system control measures (temperature and biocide levels); (iii) other risk factors (e.g., stagnation and corrosion); (iv) cleaning and disinfection practices; (v) surveillance, monitoring, and recordkeeping; and (vi) additional water systems (e.g., spa pools, cooling towers, fountains). Each item was scored as “Yes,” “No,” or “Not applicable,” with accompanying comments. Noncompliance items were noted for corrective actions (Table S1).

#### 2.7.2. Greek National Public Health Authority (NPHA) Legionella Prevention and Management Checklist [27]

This adapted checklist, originally developed for the 2004 Athens Olympic Games and subsequently incorporated into national guidelines, covers critical control points across the facility’s water network. It includes general infrastructure, cold- and hot-water systems, water-heating and storage appliances, faucets, and fire protection systems. Critical control points (CCPs) were weighed (3 points) and other non-compliance (−1 or −2 points), with the final score classified as satisfactory (A), relatively satisfactory (B), or unsatisfactory (C) (Table S2).

#### 2.7.3. Structural and Water System Risk Scoring Tables [14]

These tables attribute quantitative risk scores to structural characteristics (e.g., facility classification, age, number of floors, seasonal use, and wellness facilities) and water system features (e.g., water source, recirculation type, storage tank use, temperature profile, and cleaning protocols). Scoring was based on published literature linking these parameters to the *Legionella* colonization risk (Tables S3 and S4).

All three checklists were completed during a site inspection by trained public health officers in May 2025. Structural and operational data were cross-checked against maintenance logs, microbiological monitoring records, and direct physical inspections.

### 2.8. Risk Assessment and Statistical Analysis

Facility-specific risks were derived from the structural, operational, and physicochemical conditions. Data was analyzed using IBM SPSS Statistics v30.0 (IBM Corp., Armonk, NY, USA), Epi Info v7.2.7.0 (Centers for Disease Control and Prevention, Atlanta, GA, USA), and the MedCalc relative risk calculator statistical software free online version (MedCalc Software Ltd., Ostend, Belgium; available at https://www.medcalc.org/calc/relative_risk.php; accessed on 13 October 2025) [64,65].

Descriptive statistics included frequencies, proportions, and 95% confidence intervals (CIs), using the Wilson score method. Associations between categorical variables were tested using Chi-square or Fisher’s exact test. Linear regression was used to examine trends in the *Legionella* counts and physicochemical parameters. Relative risks (RRs) were calculated for noncompliance with the temperature and chlorine thresholds. The proportion of *Legionella*-positive samples was estimated for each water source and outlet type using Wilson’s 95% CIs. CIs when n was small (e.g., single measurements) are exact Clopper–Pearson 95% intervals for proportions wide.

Multivariate logistic regression was applied to identify predictors for the following three outcomes: (i) environmental samples positive for *Legionella* spp. (≥50 CFU/L), (ii) samples with mean concentrations ≥ 1000 CFU/L, and (iii) confirmed Legionnaires’ disease cases linked to the facility. Independent variables included facility type, water system (hot vs. cold), sampling year, hot water temperature (<50 °C vs. ≥50 °C), and free residual chlorine (<0.2 mg/L vs. ≥0.2 mg/L). Odds ratios (ORs) with 95% confidence intervals (CIs) and *p*-values were reported.

Statistical significance was defined as *p* < 0.05, and highly significant results were defined as *p* < 0.0001.

## 3. Results

### 3.1. Descriptive Epidemiology of the Confirmed Cases

In the context of a comprehensive epidemiological evaluation of LD on Crete’s island, 18 confirmed cases were documented between January and September 2025. These cases were associated with 16 hotels across the island, including two clusters, and are presented here to provide context for the outbreak investigation at the hotel under study.

The median age of the patients was 68 years (range: 43–82), with a gender distribution of 12 males (66.7%) and 6 females (33.3%). The majority of cases occurred in June (50.0%), followed by May (33.3%), while single cases were recorded in April (5.6%), August (5.6%), and September (5.6%). The median incubation period was 11 days (range: 4–23 days), and the median duration of stay was 9 days (range: 1–28 days).

Among all patients, 13 recovered (72.2%), four had unknown outcomes, and one fatality was recorded. Most cases were diagnosed through urinary antigen detection of *L. pneumophila* SG1, while two were confirmed by PCR. Notably, two patients had stayed in multiple hotels located in different regional units of the island, complicating the environmental investigation and source attribution.

**Figure 1:**
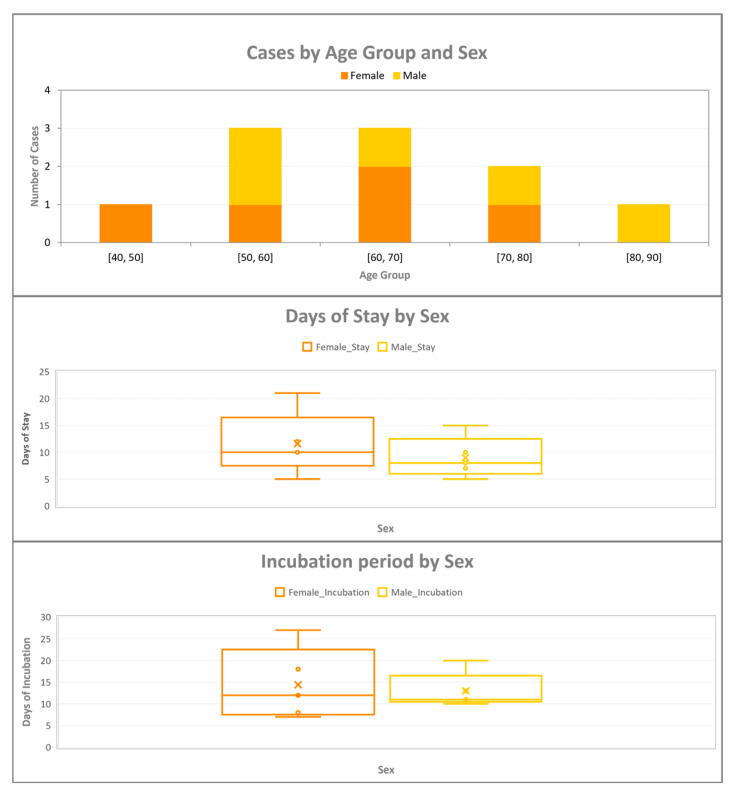
Distribution of Legionnaires’ disease cases according to demographic and epidemiological characteristics. The legend order has been revised to correspond to the color sequence of the stacked bars. The top panel illustrates the age–sex distribution of cases, the middle panel presents the duration of stay by sex, and the bottom panel depicts the incubation period by sex.

Within this broader context, the present case study focuses on a single tourist facility where 10 confirmed LD cases were linked between 2016 and 2025. The median age of the patients was 65.8 years (range: 43–81), with an equal sex distribution (5 males, 5 females) (Figure 1). The majority of cases occurred in 2025 (n = 6), with two cases in 2023 and single cases in 2017 and 2016. All patients were diagnosed using urinary antigen detection tests.

The mean incubation period was 13.0 days, while the mean exposure duration (i.e., length of stay) was 8.8 days. Eight patients recovered, whereas two had unknown outcomes due to lack of follow-up.

Analysis of the exposure characteristics revealed sex-related differences (Figure 1). The median duration of stay was longer among females (12 days, IQR: 11–12, range: 5–21) than among males (7 days, IQR: 6–8, range: 0–10). Conversely, the incubation periods were more variable in females (median: 12 days, IQR: 9–16, range: 7–27), whereas they were shorter and more consistent in males (median, 11 days; IQR, 11–12; range, 10–13).

Linear regression analysis indicated a weak positive association between the duration of stay and the number of reported cases, suggesting that longer stays may increase the cumulative exposure to aerosolized *Legionella* from showers, taps, or recreational water features. However, owing to the small sample size (n = 10) and limited variation in the exposure duration, this association should be interpreted with caution.

In addition to the temporal distribution of cases, significant variability was observed in the interval between symptom onset and official notification. The delay between symptom onset and case reporting to public health authorities ranged from 12 to 103 days (median: 30.5 days; mean: 33.2 days). This variability reflects the multi-step notification process involving both national and international public health systems. In most instances, notifications were issued approximately one month after symptom onset, potentially impacting the timeliness and accuracy of subsequent environmental investigations.

### 3.2. Microbiological Results

The analysis of the temporal distribution of *L. pneumophila*-positive samples between 2016 and 2025 demonstrated marked variability across the study period (Table 2). No positive samples were detected in 2016, 2018, 2023, or 2024. The highest positivity rates were recorded in 2017 (31% at ≥50 CFU/L, 95% CI: 7.3–49.2; 20.7% at ≥1000 CFU/L, 95% CI: 8.0–39.7 and again in 2025 12.71%, 95% CI: 7.86–17.56 at ≥50 CFU/L and 6.08% 95% CI: 2.60–9.56 at ≥1000 CFU/L.

In 2025, a notable reduction in positivity was evident after the implementation of corrective measures. Prior to the intervention, 14 out of 68 samples (20.59%; 95% CI: 12.0–32.5) surpassed the ≥50 CFU/L threshold, with 5 out of 68 (7.35%; 95% CI: 3.18–16.09) exceeding ≥1000 CFU/L. Following the intervention, all samples tested negative (0/32; 0.0–10.9%, 95% CI).

Overall, of the 181 samples collected during 2016–2025, 12.71% were positive at ≥50 CFU/L (95% CI: 7.86–17.56) and 6.08% at ≥1000 CFU/L (95% CI: 2.60–9.56). These findings underline both the temporal clustering of *Legionella* detection and effectiveness of corrective actions applied in 2025.

**Figure 2:**
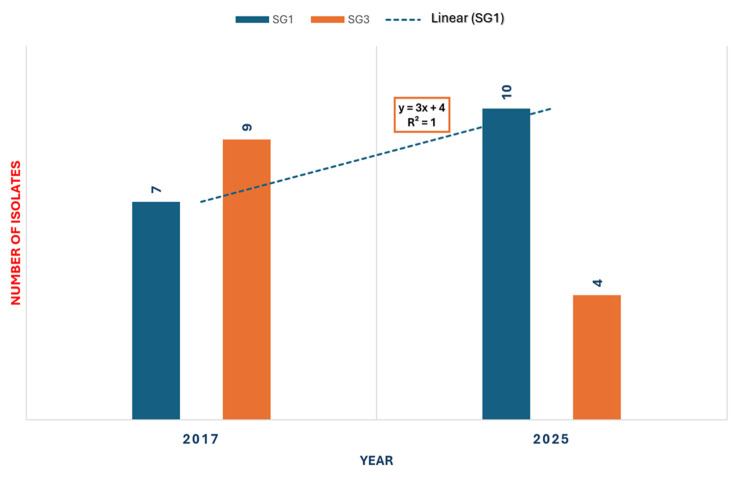
Distribution of *L. pneumophila* SG1 and SG3 isolated in 2017 and 2025. The dashed blue line represents the linear trend for SG1 isolates.

Among the 23 *L. pneumophila*-positive environmental samples collected between 2017 and 2025, *L. pneumophila* SG1 (SG1) was the most frequently detected, accounting for 56.66% (n = 17, 95% CI: 37.4%–74.5%), followed by *L. pneumophila* serogroup 3 (SG3), representing 43.33% (n = 13, 95% CI: 25.5%–62.6%). Non-*pneumophila* species were detected infrequently, comprising 13.13% (n = 4, 95% CI: 3.8%–30.7%) of positive samples. SG1 was observed in two distinct years, 2017 and 2025, with seven and ten isolates, respectively. Notably, SG1 was the predominant strain during the outbreak year of 2025, representing 71.42% of all positive isolates. SG3 was identified in 2017 (n = 9) and again in 2025 (n = 4). Overall, SG1 demonstrated temporal clustering and predominance across the study period, particularly during the outbreak year, followed by SG3. These findings highlight the dominance of SG1 within the facility’s water system over the observed years (Figure 2). During the environmental investigation, non-*pneumophila* strains were infrequently isolated, specifically in 2018 (n = 1) and 2025 (n = 3), and were not epidemiologically associated with the reported TALD cases (max = 22,000 CFU/L; min = 100 CFU/L). These findings are presented to document the overall microbial profile of the investigated water systems and to illustrate the occasional presence of non-*pneumophila* species within hotel environments.

Microbiological analysis of 16 water samples collected between April and June 2025 revealed variable total viable counts (TVC) at both 22 °C and 37 °C (Table S5). The highest heterotrophic bacterial loads at 22 °C were detected in the incoming municipal water supply (123 cfu/mL) and drinking water from a guest room tap (45 cfu/mL). At 37 °C, elevated counts were observed in the municipal supply inlet (108 cfu/mL) and indirectly at the incoming municipal supply (108 cfu/mL).

All samples tested negative for *Escherichia coli*, intestinal enterococci, and *P. aeruginosa*. Nevertheless, the kitchen sink (vegetable processing/hand washing) exhibited extremely high levels of coliform bacteria (17,000 cfu/100 mL), significantly surpassing acceptable limits and suggesting potential fecal contamination or biofilm formation. Conversely, *Legionella* spp. was identified at three sampling points: drinking water from a guest room tap (23 May 2025), boiler outlet from the collector (22 June 2025), and ice machine water (22 June 2025). These positive detections, originating from both hot- and cold-water systems, indicate the potential for extensive colonization. Although the indicator organisms generally complied with the standards, the presence of opportunistic pathogens at multiple outlets underscores the residual risk within the distribution system.

### 3.3. Association Between Total Coliforms and Presence of Legionella

Logistic regression analysis demonstrated a positive relationship between the total coliform concentration and the probability of *Legionella* detection in the environmental water samples. The predicted probability of *L. pneumophila* presence increased with rising coliform counts, with the steepest slope observed at higher values. However, several *Legionella*-positive samples were identified at very low or undetectable coliform levels, indicating that the absence of coliforms does not guarantee the absence of *Legionella*. Detailed results of the combined physicochemical and microbiological analyses of the selected samples are provided in Table S5.

### 3.4. Whole Genome Sequencing of Environmental Isolates of L. pneumophila

**Figure 3:**
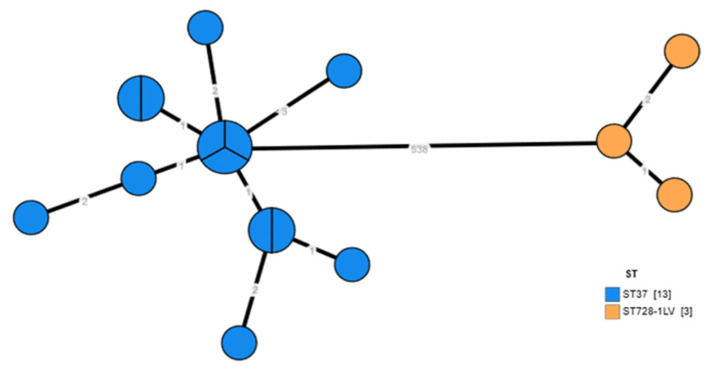
Minimum spanning tree of the 16 *L. pneumophila* isolates based on 1521 core loci. Each node represents either a single or a collection of identical isolates, color coded with their corresponding SBT sequence types. Allelic differences are indicated on each branch.

In total, sixteen 16 L. pneumophila isolates obtained from the positive points of the hotel investigated from 2025 were subjected to Whole Genome Sequencing. Thirteen isolates were assigned to SG1 and identified as sequence type (ST) 37 by sequence-based typing (SBT). The remaining three (3) isolates were identified as serogroup 3 (sg3) and all belonged to a novel sequence type not previously reported in the ESGLI SBT database. In the minimum spanning tree (MST) (Figure 3) which is based on the cgMLST, both sg1/ST37 and sg3 isolates formed two distinct tight clusters by >500 allelic difference, indicating that they represent a genetically divergent lineage. Each cluster had ≤3 allelic differences between its isolates, signifying the presence of two tight clusters with no evidence of sub-structuring among them, probably meaning that the water pipeline of the hotel was colonized by specific STs.

### 3.5. Physicochemical Parameters

**Figure 4:**
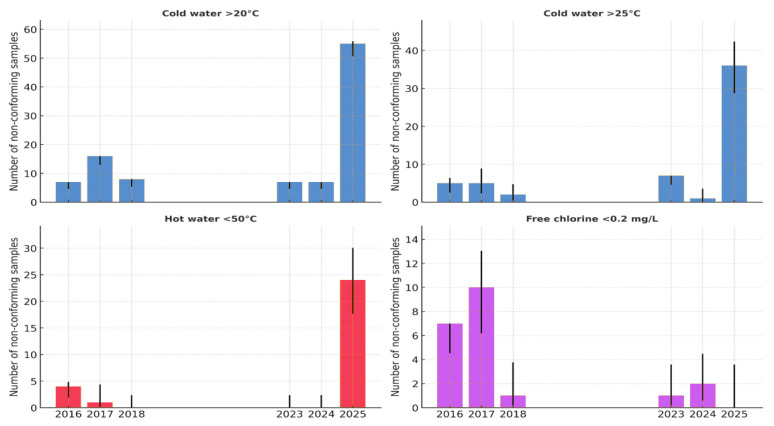
Proportion of water samples not meeting the recommended physicochemical parameters (cold water > 25 °C and >20 °C, Hot water < 50 °C and free chlorine < 0.2 mg/L) in the tourist facility during the outbreak investigation (2016–2025), with exact 95% binomial confidence intervals.

On-site measurements of the water temperature and free residual chlorine were performed for all 181 samples at the time of collection (Figure 4).

The proportion of hot water samples below the recommended threshold of 50 °C was very low in most years (≤5%) but increased sharply in 2025, when almost all samples (>95%) failed to reach the required temperature. This represents a substantial deterioration in hot-water management and creates conditions that are highly conducive to *Legionella* proliferation. At the boiler outlet, 11 targeted measurements demonstrated that only two (18.2%; 95% CI: 2.3–51.8%) achieved ≥60 °C, while the majority (9/11; 81.8%; 95% CI: 48.2–97.7%) were non-compliant. In contrast, the return of hot water temperatures (n = 9) showed partial compliance, with five (55.6%; 95% CI: 21.2–86.3%) at ≥50 °C and four (44.4%; 95% CI: 13.7–78.8%) below this threshold. These findings indicate that although circulation and reheating are partially effective, insufficient boiler outlet temperatures represent the principal systemic weakness in hot water management.

In the early years (2016–2018), all cold water samples exceeded 20 °C, while the proportion above 25 °C ranged from 25% to 71%. No data were available for 2019–2022. After 2023, a marked increase in thermal risk was observed, with 100% of the samples in 2023 and 2024 above 20 °C and 14–100% exceeding 25 °C. In 2025, nearly all samples (98%) were above 20 °C and 64% exceeded 25 °C. These findings indicate a progressive loss of cold-water temperature control and widespread conditions favorable for *Legionella* proliferation.

Between 2016 and 2018, 30–50% of the samples showed insufficient residual disinfectant (<0.2 mg/L), with wide annual variability. Although the levels improved after 2020, deficiencies persisted in 2023–2024, with up to ~10% of samples below the recommended threshold.

### 3.6. Association Between Physicochemical Non-Compliance and Legionella Positivity

Preliminary univariable analyses examined the relationship between physicochemical noncompliance and *Legionella* positivity (≥50 CFU/L) (Table 3). Hot water below 55 °C was associated with a higher likelihood of positivity (RR = 1.81, 95% CI: 0.75–4.35), whereas free chlorine levels below 0.2 mg/L (RR = 0.33, 95% CI: 0.05–2.23) were associated with a lower observed risk. None of these associations reached statistical significance, reflecting sparse data or wide confidence intervals.

Hot water < 55 °C was recorded in 17/80 hot water measurements (21.3%), cold water > 20 °C in 100/181 cold water measurements (55.2%), and free chlorine < 0.2 mg/L in 21/80 measurements (11.6%).

These exploratory results representing that suboptimal hot water temperatures (<55 °C) may increase the likelihood of *Legionella* colonization, consistent with the established risk mechanisms. The apparently “protective” associations observed for cold water > 25 °C and low chlorine are likely due to the limited number of positive samples and confounding effects. Larger datasets are required to confirm or refute these associations (Table 3).

### 3.7. Chemical Composition of Selected Water Samples

Chemical analysis of 11 sampling points conducted from April to June 2025 showed values largely within the international drinking water standards, with some notable elevations. The pH ranged from 7.4 to 8.0 (measured at 25 °C), which is within the WHO guideline range. The chloride concentrations were elevated (350–371 mg/L), particularly at the cold tap in the departure area, whereas the conductivity was consistently high (1050–1419 µS/cm at 20 °C) across all points tested. The water hardness ranged from 15 to 21.7 °dH, classifying the supply as moderately hard to hard. Turbidity reached 139 NTU at the cold tap in the departure area, although nitrates and nitrites remained well below the EU’s maximum permissible values.

**Figure 5:**
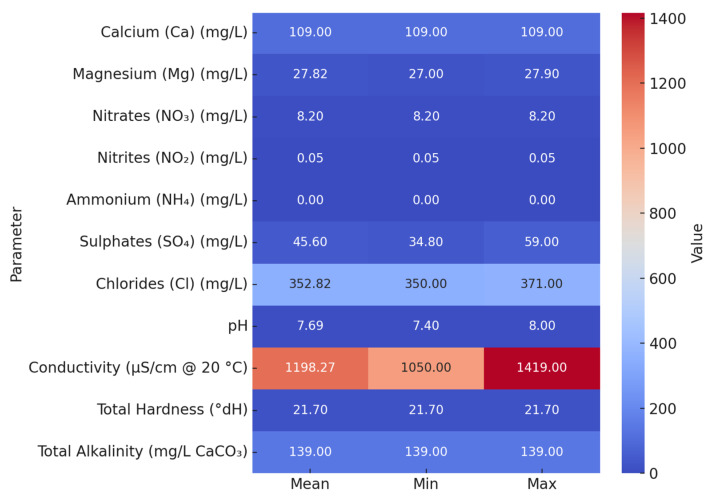
Summary statistics (mean, minimum, and maximum) of physicochemical parameters measured in water samples collected from the tourist facility between April and July 2025.

The distributions of the chloride, conductivity, and hardness values across the sampled outlets are shown in Figure 5. Overall, the results indicated a mineral-rich composition and uniformity in the quality of the municipal water supply across the distribution network. Elevated hardness and conductivity are of particular relevance to *Legionella* risk assessment, as they favor scale formation and biofilm development, conditions that may protect *Legionella* from disinfection and promote persistence. Although only a limited number of water samples were subjected to chemical testing, their mean values were considered representative of the overall system, given a single, stable municipal source.

### 3.8. Dead Ends and Stagnant Lines Risk Assessment

Eighteen points with potential for water stagnation were identified within the hotel’s water distribution system, comprising five dead ends (27.8%) and 13 stagnant lines (72.2%). The majority of stagnation points were located on the ground floor (n = 11; 61.1%), followed by the basement (n = 3; 16.7%), fifth floor (n = 3; 16.7%), and terrace (n = 1; 5.6%). Ground-floor hotspots were primarily situated in restaurant, bar, and guest room areas, highlighting the risk of stagnation across both public and private water outlets. Stagnant points were associated with fire hose reels (n = 2; 11.1%), both connected to the potable water supply; showers (n = 3; 16.7%), all in unused or under-construction areas; sinks/taps (n = 4; 22.2%), some of which were inaccessible due to equipment placement; and WCs/bidets (n = 4; 22.2%), all located in unused rooms. Additional isolated points include one washing machine, one post-mix beverage supply line, one solar panel supply line, and two kitchen connections without terminal fixtures.

### 3.9. Multi-Tool Risk Assessment Approach

#### 3.9.1. ECDC Facility Inspection Tool for Travel-Associated Legionnaires’ Disease for Reducing Legionella Risk

A structured evaluation of the facility’s control practices was conducted using the European Technical Guidelines (2017) checklist (Table S1). Of the six assessed domains, four demonstrated complete noncompliance, while two showed only partial compliance. None of the domains achieved complete compliance (Table 4).

Key deficiencies include the absence of a designated responsible person, lack of staff training, and absence of a written control program or logbooks. Preventive practices such as systematic flushing of outlets and maintenance of showerheads were not carried out, and stagnant pipework with visible corrosion was identified. Temperature monitoring of the hot water systems only began in late May 2025 and thus did not represent continuous surveillance. Biocides were utilized (chlorine dioxide (obtained as a stabilized solution) in 2025, hydrogen peroxide previously), but no evidence of monitoring residuals at the distal outlets was available. The calorifiers and tanks were cleaned, but no standard operating procedures were documented. Although no spa pools or cooling towers were present, other secondary water systems (outdoor showers, irrigation, and decorative fountains) existed on site without any evidence of routine monitoring.

#### 3.9.2. Greek National Public Health Authority (NPHA) Legionella Prevention and Management Checklist

The standardized checklist assessment (Table S1) yielded a total score of 38, corresponding to an unsatisfactory result (Category C) as it exceeded the >20% threshold of the maximum negative score and included multiple critical violations (Table S2). Key deficiencies in several domains have been identified.

Sediments were present in the storage tanks, and annual cleaning/disinfection was not documented. Unused taps and stagnant piping without systematic removal or flushing were observed. Showerheads and taps were not regularly descaled, and appliances were not consistently maintained under sanitary conditions.

No comprehensive logbooks were available for temperature, chlorine, or microbiological monitoring. Random water checks were not systematically performed, and there was no documented evidence of negative *Legionella* results in the preceding six months. Cold water was not consistently maintained below 25 °C, hot water failed to reach >50 °C within 2 min at the outlets, and chlorine residuals were frequently <0.2 mg/L.

#### 3.9.3. Structural and Water System Risk Scoring Tables

The structured risk assessment of the facility identified multiple vulnerabilities at both the structural and water system levels (Tables S3 and S4).

The facility reached a cumulative risk score of 30 points, reflecting several high-risk characteristics. Notably, no WSP was implemented, and no designated personnel were responsible for *Legionella* control. The facility was classified as a <4–5 star hotel, >20 years old, >10 rooms per floor, and >40% of rooms with showers, all of which contributed to elevated scores due to increased building complexity and aged plumbing. Seasonal operation, absence of automated chlorination, and location in a small municipality (<10,000 residents) further increased structural risk.

The water system scored 36 points, indicating widespread deficiencies. The key factors included mixed water sources, partial recirculation, use of storage tanks, and absence of upstream treatment before water heaters. Preventive practices were limited; there was no formal cleaning and disinfection protocol, no systematic inspection or disinfection of storage tanks, and no registers, calendars, or checklists for documenting maintenance. The showerheads and taps were not replaced on a scheduled basis, and microbiological monitoring for *Legionella* was not routinely performed.

Taken together, the combined total structural (30/52) and water system (36/71) risk scores highlight a facility operating at the maximum documented risk level with multiple overlapping deficiencies in design, operation, and maintenance that significantly increase the likelihood of *Legionella* colonization and transmission.

**Figure 6:**
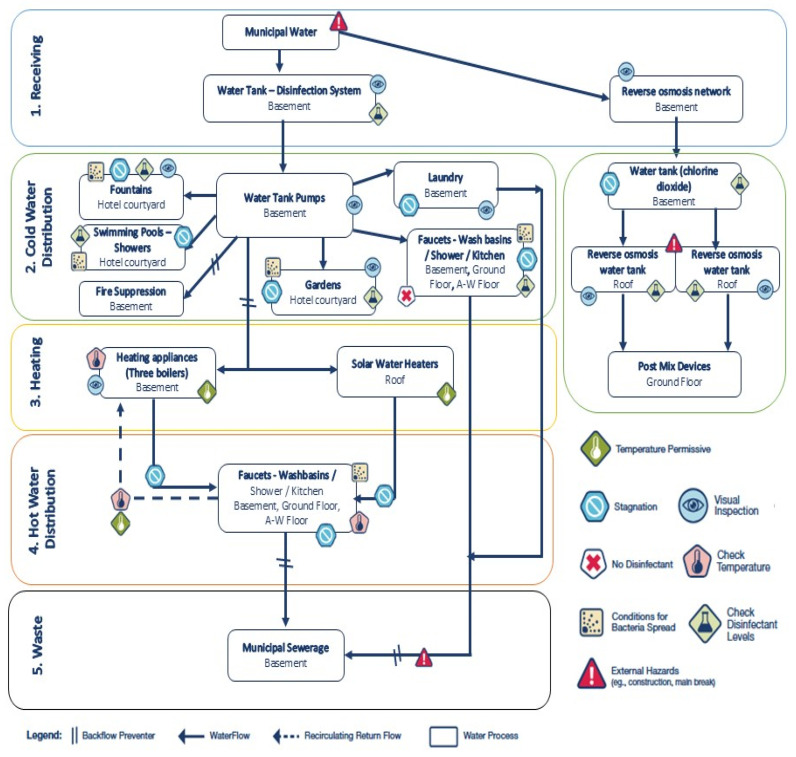
Flowchart of the hotel’s water distribution system based on the freely available CDC manual “Developing a Water Management Program to Reduce *Legionella* Growth & Spread in Buildings: A Practical Guide to Implementing Industry Standards.”

Finally, the facility’s water distribution system was mapped according to the CDC Water Management Toolkit methodology (Figure 6). The schematic highlights multiple risk points for *Legionella* growth and spread, including stagnation areas, storage tanks, solar water heaters, and recirculating loops within permissive temperature ranges. The absence of disinfectants and external hazards (e.g., main breaks and construction) was also noted. Control measures, such as disinfectant monitoring, temperature checks, and visual inspections, were embedded into the schematic to provide a comprehensive framework for prevention and mitigation. Together, the high-risk scores and the measured deficiencies in temperature control, biocide residuals, and hydraulic design provide convergent evidence of a facility environment highly favorable for *Legionella* proliferation and persistence.

**Figure 7:**
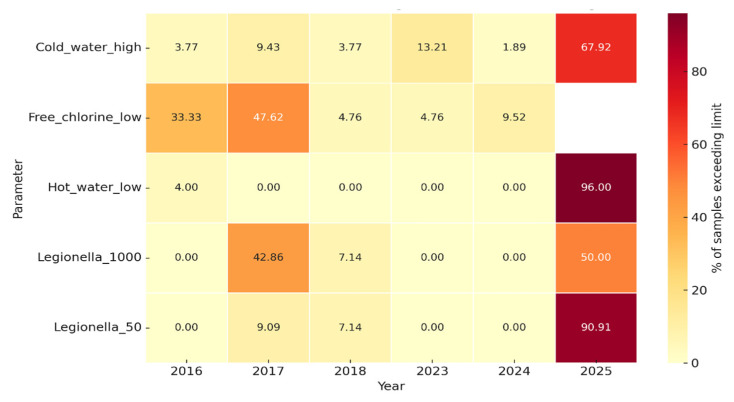
Heatmap showing the percentage of water samples exceeding the control limits for *Legionella* load (≥50 CFU/L and ≥1000 CFU/L), free chlorine (<0.2 mg/L), cold-water temperature (>25 °C), and hot-water temperature (<50 °C) in the investigated hotel.

A heatmap summary of environmental non-compliance is shown in Figure 7, illustrating annual exceedances across microbiological (*Legionella* thresholds) and physicochemical parameters (temperature and free chlorine). Peaks in noncompliance were observed in 2017 and 2025, coinciding with the occurrence of clinical cases.

**Figure 8:**
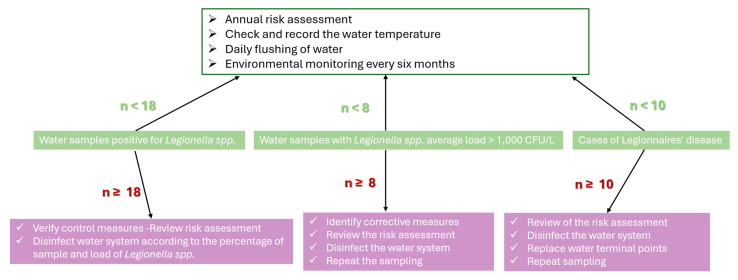
Decision Tree for Corrective Actions by Hotel Administration Based on Identified Risk Triggers [1].

In line with the European Technical Guidelines, corrective measures were linked to predefined monitoring thresholds (Figure 8) [14]. Escalation was required when ≥10 LD cases occurred, ≥8 samples exceeded 1000 CFU/L, or ≥18 samples were positive, regardless of the load. Actions included system-wide disinfection, replacement of terminal outlets, review of RA, and repeat monitoring. Lower-level detection (<8 samples ≥1000 CFU/L or <18 positives overall) required verification of existing control measures and targeted disinfection.

## 4. Discussion

This case study highlights a significant public health concern within hotel environments, demonstrating the potential for outbreaks to occur in the absence of systematic, risk-based control measures, even years after initial disease clusters and following extended periods of negative environmental testing for *Legionella*. The findings underscore the importance of continuous environmental surveillance and preventive maintenance, as the intermittent or seasonal operation of tourist facilities may facilitate the persistence of *L. pneumophila*. The re-emergence of cases in the same accommodation setting after transiently successful remediation efforts illustrates the challenges in achieving sustainable pathogen control and emphasizes the need for systematic risk assessment approaches in hotel water systems.

In accordance with European legislation, risk assessments are mandated to safeguard human health. The Drinking Water Directive (EU 2020/2184) requires a risk-based approach across the entire drinking water supply chain—from catchment areas to the tap—with Member States expected to complete their first comprehensive assessments by 2026 [66,67]. Similarly, the “WHO Guidelines for Drinking-Water Quality” advocate for the implementation of WSPs as the most effective strategy for consistently ensuring the safety and acceptability of drinking-water supplies. The WSP approach constitutes WHO’s health response to the Sustainable Development Goal (SDG) 6, which aims to secure safe drinking water for all [68].

These challenges are not confined to Crete but reflect a broader pattern observed in southern European tourist destinations. In Mediterranean tourist areas, similar findings have been observed, for instance in Sardinia with colonization rates of 61.3% [69], in southern Italy during the period 2019–2021 with increased contamination [70,71], and 65.4% of the tourist facilities associated with suboptimal temperatures and chlorine levels [72].

Microbiological thresholds alone inadequately reflect the *L. pneumophila* infection risk. Resistance mechanisms, such as biofilm development and survival within amoebae, increase persistence despite disinfection [73]. Moreover, exposure can occur through both aspiration and inhalation pathways, which are not captured by culture thresholds [73,74]. Quantitative microbial risk assessment (QMRA) models show that the concentration variability and exposure dynamics better explain transmission potential than the absolute culture results [75,76,77].

This study investigated an outbreak of LD associated with a single tourist accommodation in Crete in 2025. Ten confirmed cases have been associated with their stay at this hotel in total, two in 2016–2018 and eight in 2023–2025. When compared with the broader epidemiological profile of 18 confirmed cases reported across Crete in the same year, this establishment alone accounted for one-third (6/18) of all cases, underscoring its epidemiological importance and the persistence of local risk factors. The recurrence of cases over several years despite previous control actions proposes that the hotel’s preventive measures were insufficient to achieve long-term elimination of the pathogen.

The observed reporting delay (median: 30.5 days, range: 12–103 days) highlights a critical limitation in the surveillance of TALD. The multi-level reporting pathway—spanning clinicians, national authorities, and international networks—introduces significant temporal gaps between exposure and environmental sampling. This lag may hinder source identification, as environmental conditions often change substantially during this interval.

### 4.1. Epidemiological Characteristics of Cases

On the island of Crete, a total of 18 confirmed cases were reported across 16 hotels as of September 2025, with one additional hotel experiencing clusters—the facility investigated in this case study. The majority of cases experienced symptom onset in June (50%), followed by May (33.3%) and April (19%). This pattern coincides with the reopening of hotels and the reduced occupancy at the onset of the tourist season following the winter closure, as most hotels do not operate year-round. This observation is consistent with other studies indicating that stagnation in water systems during periods of non-operation or low occupancy facilitates the colonization of *Legionella* [77,78,79].

The median age of the patients was 68 years (range: 43–82), and two-thirds were male (66.7%), highlighting the increased vulnerability of older men, consistent with the European epidemiological patterns of LD. The incubation periods, ranging from 4 to 23 days (median: 11 days), correspond to the expected variability of TALD cases. According to the ELDSNet and ECDC case definitions, the incubation period for LD typically ranges from 2 to 10 days, most commonly 5–6 days, and careful alignment of exposure dates with symptom onset is critical to determine the most feasible site of infection [13,35,45,47,49,80]. Most patients recovered (72.2%), while four had unknown outcomes and one fatality was recorded, reflecting differences in follow-up availability and medical reporting.

Two patients had a history of staying in multiple accommodation facilities in different regional units. This highlighted the need for molecular typing to confirm potential epidemiological identifications between clinical samples and environmental isolates.

According to the epidemiological data, the hotel under study accounted for approximately 37.5% of all cases reported in Crete in 2025. A total of 10 confirmed cases of TALD were linked to this facility between 2016 and September 2025, indicating a recurrent occurrence of LD within the same accommodation setting.

The median patient age (65.8 years) was comparable to that of all reported cases in Crete (68 years) and consistent with previous findings showing that older adults with underlying conditions represent the most susceptible group [80,81,82].

Unlike the typical male predominance observed across Europe (male-to-female ratio > 2:1) [83,84,85], and in the overall 2025 Crete dataset (66.7% male), the cases in this facility were equally distributed by sex. Female patients tended to have longer stays and more variable incubation periods, whereas male patients developed disease after shorter and more consistent exposures. These findings suggest that cumulative exposure during prolonged stays may increase infection risk in females, whereas shorter, high-intensity exposures may suffice for males—a pattern consistent with studies identifying stay duration as a risk modifier [86,87,88].

The mean incubation period of 13.0 days falls within the expected 2–14-day range, although the broader variability among females mirrors the reported outliers in travel-associated cases [89,90]. Importantly, six cases occurred within a three-week period in 2025, despite relatively low environmental positivity, demonstrating that transient amplification events can trigger outbreaks that exceed predictions based solely on environmental monitoring [91,92].

Overall, the demographic profile of the affected guests (median age 65.8 years, equal male-to-female ratio) reflects exposure to shared environmental sources rather than behavioral differences. The longer duration of stay among female patients may have increased their cumulative exposure, while the observed association between exposure time and disease occurrence supports a dose-dependent risk model. However, the small sample size limits the statistical strength of these observations.

The temporal distribution of cases, concentrated in spring and early summer, coincides with the reopening of hotels after winter closure, when stagnation and temperature fluctuations in the water systems favor *Legionella* growth. This seasonal pattern mirrors the island-wide trend, reinforcing that periods of low occupancy and system inactivity represent critical risk windows requiring targeted preventive action.

Given that the present investigation was completed in September 2025, the winter closure period was not assessed in detail. However, it should be noted that this period may also contribute to an increased risk of *Legionella* proliferation, particularly due to reduced system operation and insufficient hot-water temperatures, especially in facilities relying primarily on solar water heating systems [93,94,95].

### 4.2. Environmental Positivity and Threshold Limitations

This investigation highlights that low culture-based positivity is not synonymous with low transmission risk. *Legionella* spp. were detected in only 13.81% of the samples, with 7.73% above the ≥1000 CFU/L threshold (Table 2), yet a substantial 2025 cluster occurred. Similar patterns have been described in Greece and across southern Europe, where clusters emerged despite limited environmental positivity [91,92,96,97,98]. The temporal clustering of cases in May 2025, coinciding with rising temperatures and fluctuating water demands, supports a transient amplification event. Taking immediate measures—including thermal disinfection, chlorine dioxide dosing, and flushing—likely curtailed further cases in summer 2025. These findings restate the limitations of relying solely on microbiological thresholds as risk proxies.

### 4.3. Spatial Distribution

The predominance of *L. pneumophila* SG1 (Figure 2) is consistent with its global epidemiological importance [21,99,100]. SG1 accounted for most positive samples during the outbreak year, coinciding with confirmed cases, and exhibited a slight increasing trend over the study period, as indicated by the linear trend line, which could potentially explain the occurrence of the outbreak. The detection of SG3 and non-*pneumophila* species, though less frequent, highlights the diverse colonization potential of hotel water systems and the need for surveillance beyond SG1 [94,101]. Non-*pneumophila* species were observed only sporadically and were not associated with TALD cases; however, their inclusion provides a comprehensive characterization of the *Legionella* population within the facility.

Our previous research, based on environmental surveillance conducted in hotels across Crete between 2020 and 2025, confirmed the persistent colonization by *L. pneumophila*, specifically SG1 (29.60%, 95% CI: 20.7–40.6%) and SG2-15 (72.53%, 95% CI: 62.4–81.1%). Although *L. pneumophila* SG1 was detected less frequently than SG2-15, it remains of particular epidemiological importance, as it is the primary cause of LD worldwide. This finding aligns with previous reports, as Kowalczyk et al. (2023) identified that *L. pneumophila* SG2–15 are more prevalent in building water systems compared to *L. pneumophila* SG1. [102,103,104]. Notably, *L. pneumophila* SG1 was most often detected in systems with hot water temperatures below 50 °C and residual chlorine concentrations below 0.2 mg/L [41,95].

### 4.4. Physicochemical and Structural Risk Factors

The statistical analysis of the physicochemical parameters indicated systemic deficiencies in the management of the required high water temperatures of the hotel’s water system, which contribute to its colonization by *Legionella*. The measurements of the temperatures of the hot water leaving the boiler for distribution were in the majority below 60 °C, and the temperatures of the hot water returning to the engine room for reheating in the boiler were below 50 °C. This was a particularly worrying finding and in complete contrast to the internationally accepted requirements. Boiler outlet temperatures are critical not only for thermal disinfection but also for ensuring that hot water, even to the most remote point of the installation, is delivered at least 50 °C. Although the hot water circulation demonstrated partial compliance, these measures were insufficient to compensate for the low outlet efficiency. This finding is consistent with evidence that inadequate heating is a significant risk factor. Monitoring of cold-water temperatures revealed persistent non-compliance after 2023, with all samples exceeding 20 °C and a substantial proportion exceeding 25 °C (up to 64% in 2025). These temperatures fall within the optimal growth range for *Legionella* and indicate deficiencies in maintaining adequate thermal control, potentially due to inadequate insulation, stagnation, or systemic design limitations. This trend may also be exacerbated by the ongoing climate crisis, which has led to elevated ambient temperatures in the Mediterranean basin. The progressive decline in cold-water quality underscores the critical need for effective thermal management in both hot and cold distribution networks, as failure in either system facilitates *Legionella* colonization and persistence [41].

Free residual chlorine concentrations showed partial improvement after 2020 but remained intermittently deficient, with up to 10% of samples below 0.2 mg/L in 2023–2024. While disinfectant monitoring is an essential component of control, the present findings reinforce that chemical treatment alone cannot compensate for insufficient thermal management. Sustained microbial risk reduction requires both adequate disinfection and compliance with the thermal thresholds. The exploratory risk analysis supports the established role of thermal conditions in *Legionella* ecology.

Elevated chloride, conductivity, and hardness levels indicated a mineral-rich groundwater supply (Figure 5), favoring scale deposition and biofilm development that reduce the disinfectant efficacy [105,106,107]. Consistently low residual chlorine levels across multiple outlets suggest systemic issues, indicating that the problem is not limited to individual fixtures but reflects broader characteristics of the water supply system—such as insufficient disinfection or inadequate dosing practices. Structural vulnerabilities, such as blind ends, stagnant lines, and complex recirculation loops, further promoted colonization despite generally low culture positivity. Seasonal operation intensifies these risks, as prolonged stagnation during closure periods fosters microbial regrowth [26,78,108]. Facilities with ratings below four stars may experience increased vulnerability due to limited resources, outdated plumbing systems, and suboptimal implementation of WSPs [108,109]. In this case, the facility scored 30/52, indicating a moderate to high structural risk profile shaped by seasonal operation and organizational limitations.

### 4.5. Microbiological Indicators and Predictive Value

The detection of exceedingly high coliform levels (17,000 cfu/100 mL) at the kitchen sink used for vegetable processing and hand washing presents significant concerns regarding hygiene and potential fecal contamination. This observation not only surpasses acceptable thresholds but also implies the possibility of biofilm formation or plumbing inadequacies. Nevertheless, the presence of *Legionella* in samples with low or undetectable coliform levels suggests that coliforms alone are not sufficient prognostic factors, highlighting the need for comprehensive monitoring that includes both microbiological and structural parameters.

### 4.6. Added Value of Structured Risk Assessment

The application of three structured risk assessment (RA) tools (Tables S1–S4), including the ECDC and Greek NPHA checklists, revealed consistent deficiencies in design, operation, and verification. Over 60% of the data in the inspection reports were non-compliant or partially compliant. Significant requirements that were not met concerned blind spots, hot water temperatures, inadequate flushing, incomplete records and the absence of controls, and finally the absence of a person responsible from the staff designated by the management for protection against *Legionella* and the training of staff directly related to this issue. Importantly, outlets with high-risk priority numbers overlapped with *Legionella*-positive sites, confirming the predictive value of RA, even when positivity rates were low. These findings support European evidence that RA reveals hidden vulnerabilities overlooked by culture alone [64,79,80,81]. The CDC Water Management Toolkit, combined with facility flowcharts and corrective action decision trees (Figure 6, Figure 7 and Figure 8), further illustrated how RA outputs can guide interventions and staff training in complex hotel water systems. These findings underline the importance of incorporating RA into hotel inspection protocols across tourist regions.

### 4.7. Comparison with Similar Cluster and Outbreak Investigations

Our findings align with European investigations showing that outbreaks can arise from either (i) localized, high-concentration sources (e.g., spas) or (ii) complex systems with intermittent colonization [83,98,110,111,112]. Borella et al. [113] linked structural complexity to risk regardless of the culture results, while De Filippis et al. [114,115] highlighted the absence of WSPs and thermal control. In our case, only three samples (1.66%) exceeded 10,000 CFU/L, yet six cases occurred—in contrast to spa outbreaks, where counts exceeded 10^6^ CFU/L [116]. This suggests that even a few contaminated outlets, when combined with systemic risks, can sustain transmission.

### 4.8. Public Health Implications

This study demonstrates that clusters may occur in facilities that are compliant with microbiological thresholds. RA integration into the surveillance and outbreak response provides a standardized evaluation, early hazard identification, and prioritization of remediation. In this case, the RA findings directly informed corrective measures, such as the removal of blind ends, chlorine dioxide dosing, and flushing protocols, aligning with the European Technical Guidelines [2]. Notably, the facility lacked cooling towers or spas; however, a large cluster still occurred. This reinforces the fact that risk cannot be excluded in the absence of traditionally recognized high-risk systems and highlights the need for comprehensive guideline-based controls across all water systems.

### 4.9. Strengths and Limitations

The strengths of the study include the time span (2016–2018 and 2023–2025), the integration of microbiological, physicochemical and structural data, and the use of internationally recognized RA tools. The limitations include the fact that the study is based on a single facility, which may limit the generalizability of the findings. Moreover, the low levels of free chlorine observed in some samples may be attributable to the use of chlorine dioxide as a secondary disinfectant. Chlorine dioxide can react with or reduce free available chlorine (FAC), potentially leading to lower residual chlorine concentrations in the system. This interaction should be considered when interpreting chlorine measurements and assessing the disinfection effectiveness [117,118,119].

A significant limitation of this study is the inherent delay between patient exposure and the subsequent environmental sampling. This temporal gap arises from the multi-step notification process involving both international and national public health authorities, frequently resulting in investigations being conducted weeks or even months following the initial exposure. In certain instances, particularly when notifications are received after the season has concluded, assessments are postponed until the facility reopens. Furthermore, limited staffing within local public health services may intensify these delays. These factors may impact the detection of *L. pneumophila* and should be considered when interpreting environmental results. This limitation highlights the importance of not exclusively depending on environmental investigation as the primary method in LD case investigations. Moreover, the absence of isolation of *Legionella* from patient samples, the diagnosis was based on urine tests, does not allow for the definite linking of TALD cases with the suspected source of infection, since WGS was applied in *Legionella* isolated from the hotel only.

## 5. Conclusions

To mitigate the risk of *L. pneumophila* in high-risk environments such as hotels, it is imperative to first conduct a comprehensive risk assessment and then continuously monitor the internal water distribution network. The assessment should include not only the microbiological quality of water intended for human consumption, but also structural, operational and epidemiological factors. Preventive strategies should include removing dead ends and stagnant lines, separating fire extinguishing systems from water distribution networks, regularly flushing stagnant lines and continuously monitoring water temperature and disinfectant levels. It is necessary to designate and train responsible personnel or external partners, implement a Water Management System (WSP) and maintain systematic monitoring based on documented risk assessments.

Prior to the operating season, it is essential that all water distribution systems undergo a comprehensive inspection, draining and disinfection after periods of extended closure. Hot water should be maintained at ≥50 °C and cold water at ≤25 °C, with adequate concentrations of chlorine or other approved disinfectants to ensure microbiological safety. Addressing complex recirculation cycles and other potential sources of water stagnation is equally critical. Incorporating these procedures into seasonal opening protocols establishes a standardized framework for ensuring water quality and supports public health authorities in approving the reopening of hospitality facilities, while strengthening the prevention of drug overdoses. Finally, it is equally important to continue implementing these measures until the end of the tourist season, where illnesses are also occurring due to under-functioning facilities. These measures are crucial for compliance with European directives and national legislation and for reducing the incidence of FMD cases.

## Figures and Tables

**Table 1:** Hotel Water Network Characteristics.

Parameter	Description
Pipe material	PVC
Number of boilers	3 × 2000 L
Hot water circulation	Throughout the building via a circulation pump
Heating system	Gas boiler with solar panel assistance
Seasonal operation	May–October
Dead ends	5 (27.8%)
Stagnant lines	13 (72.2%)
Backflow prevention devices	Installed, compliant with NF EN 1717

**Table 2:** Annual distribution of *L. pneumophila*-positive environmental samples at two thresholds (≥ 50 CFU/L and ≥1000 CFU/L), with 2025 data shown separately for pre- and post-intervention periods.

Year	Total Samples (n)	≥50 CFU/L Positive (n, %) [95% CI]	≥1000 CFU/L Positive (n, %) [95% CI]
2016	12	0 (0.00%) [0.00–26.46]	0 (0.00%) [0.00–26.46]
2017	29	10 (31.03%) [7.3–49.2]	6 (20.69%) [9.84–38.39]
2018	14	0 (0.00%) [0.0–21.4]	0 (0.00%) [0.0–21.4]
2023	13	0 (0.00%) [0.00–24.71]	0 (0.00%) [0.00–24.71]
2024	13	0 (0.00%) [0.00–24.71]	0 (0.00%) [0.00–24.71]
2025 (Pre-Intervention)	68	14 (20.59%) [12.0–32.5]	5 (7.35%) [2.4–16.3]
2025 (Post-Intervention)	32	0 (0%) [0.0–10.9]	0 (0.00%) [0.0–10.9]
Total (2016–2025)	181	23 (12.71%) [7.86–17.56]	11 (6.08%) [2.60–9.56]

**Table 3:** Association between physicochemical noncompliance and *L. pneumophila.* positivity (≥50 CFU/L).

Parameter	*Legionella* +	*Legionella* −	Total	Relative Risk (95% CI)	*p*-Value
Hot water < 55 °C	5	12	17	1.81 (0.75–4.35)	0.20
Hot water ≥ 55 °C	10	53	63	-	–
Free chlorine < 0.2 mg/L	4	17	21	1.09 (0.41–2.92)	0.85
Free chlorine ≥ 0.2 mg/L	11	48	59	-	–

**Table 4:** Compliance with the European Technical Guidelines (2017) checklist for minimizing *Legionella* risk in building water systems at the investigated tourist facility.

Domain	Key Requirements	Compliance	Comments
1. Personnel and responsibility	Appointed responsible person; staff training; external contractors competent	No	No designated person; no staff training
2. Control measures	Potable supply; hot water 50–60 °C; cold water < 25 °C; biocides monitored	Partial	Public water supply; chlorine dioxide in 2025; temperature monitoring only from late May 2025; no biocide monitoring
3. Other risk factors	Flushing outlets; cleaning showerheads; no dead-legs; no corrosion	No	No flushing; no showerhead maintenance; stagnant pipework present; visible corrosion at outlets
4. Cleaning and disinfection	Annual calorifier/tank cleaning; seasonal disinfection; filters/softeners maintained	Partial	Calorifiers and tanks cleaned; no filters; no written SOPs; incomplete network disinfection
5. Surveillance and documentation	Written control programme; logbooks; risk assessment every 2 years; independent audit	No	No written programme, logbooks, or audit
6. Particular water systems	Spa pools; cooling towers; other high-risk systems	Not applicable	No spa pools or cooling towers; outdoor showers, irrigation and fountains present but not monitored

## Data Availability

The original contributions presented in the study are included in the article, further inquiries can be directed to the corresponding author.

## Supplementary Materials

The following supporting information can be downloaded at: https://www.mdpi.com/2076-0817/14/10/1059/s1, Table S1: Check List According to the European Technical Guidelines 2017: minimizing the risk from *Legionella* infections in building water systems, Table S2: Risk scores attributed to structural features of touristic-recreational facilities. The checklist has been adapted from the European Guidelines for Prevention and Control of Travel Associated Legionnaires’ disease and from the National School of Public Health 2004 Athens Olympic Games Checklist for building water systems, Table S3: Risk scores attributed to structural features of touristic-recreational facilities, Table S4: Risk scores attributed to water systems of touristic-recreational facilities, Table S5: Physicochemical and microbiological parameters of selected water samples collected from the hotel’s water distribution system, April–June 2025. References [14,27,94,113,115,116] are cited in the Supplementary Materials.

## Abbreviations

The following abbreviations are used in this manuscript:

LD	Legionnaires’ disease
ELDSNet	European Legionnaires’ Disease Surveillance Network
TALD	Travel-Associated Legionnaires’ Disease
ECDC	European Centre for Disease Prevention and Control
CFU	Colony-forming unit
SG	Serogroup
PCR	Polymerase chain reaction
TVC	Total Viable Count
WSP	Water Safety Plan
RA	Risk Assessment
RPN	Risk Priority Number
WMP	Water Management Program
