# Correction: Papadakis et al. Beyond Microbiological Analysis: The Essential Role of Risk Assessment in Travel-Associated Legionnaires’ Disease Outbreak Investigations. *Pathogens* 2025, *14*, 1059

**DOI:** 10.3390/pathogens14111160

**Published:** 2025-11-14

**Authors:** Antonios Papadakis, Eleftherios Koufakis, Vasileios Nakoulas, Leonidas Kourentis, Theodore Manouras, Areti Kokkinomagoula, Artemis Ntoula, Maria Malliarou, Kyriazis Gerakoudis, Katerina Tsilipounidaki, Dimosthenis Chochlakis, Anna Psaroulaki

**Affiliations:** 1 Department of Clinical Microbiology and Microbial Pathogenesis, School of Medicine, University of Crete, 71110 Heraklion, Greece; kokareti@gmail.com (A.K.); artemisntoula@gmail.com (A.N.); maria.malliarou.ger@gmail.com (M.M.); surreydimos@hotmail.com (D.C.); 2 Public Health Authority of the Region of Crete, 71201 Heraklion, Greece; 3 Civil Protection of the Region of Crete, 71201 Heraklion, Greece; elkoufakis@crete.gov.gr; 4 Department of Materials Science and Engineering, University of Crete, 70013 Heraklion, Greece; tman@iesl.forth.gr; 5 Department of Hygiene and Epidemiology, Faculty of Medicine, University of Thessaly, 41222 Larissa, Greece; nakoulasb@gmail.com (V.N.); leokourentis@uth.gr (L.K.); 6 Regional Laboratory of Public Health of Crete, School of Medicine, 70013 Heraklion, Greece; k.gerakoudis@eody.gov.gr (K.G.); tsilipoukat@gmail.com (K.T.)

There was an error in the original publication [1].

A redundant paragraph remained in the published version due to an oversight during the final editing stage.

A correction has been made to the Results Section, Paragraph 4.

The redundant text (“As of September 2025, … while two were confirmed by PCR.”) has been deleted to improve clarity.

In addition, there was a mistake in Figure 1 as published.

The figure was of insufficient resolution due to a technical issue in the export process during manuscript preparation.

The corrected Figure 1, identical in data and content but recreated in higher resolution for improved visual clarity, appears below:

The authors state that the scientific conclusions are unaffected. This correction was approved by the Academic Editor. The original publication has also been updated.

## Figures and Tables

**Figure 1:**
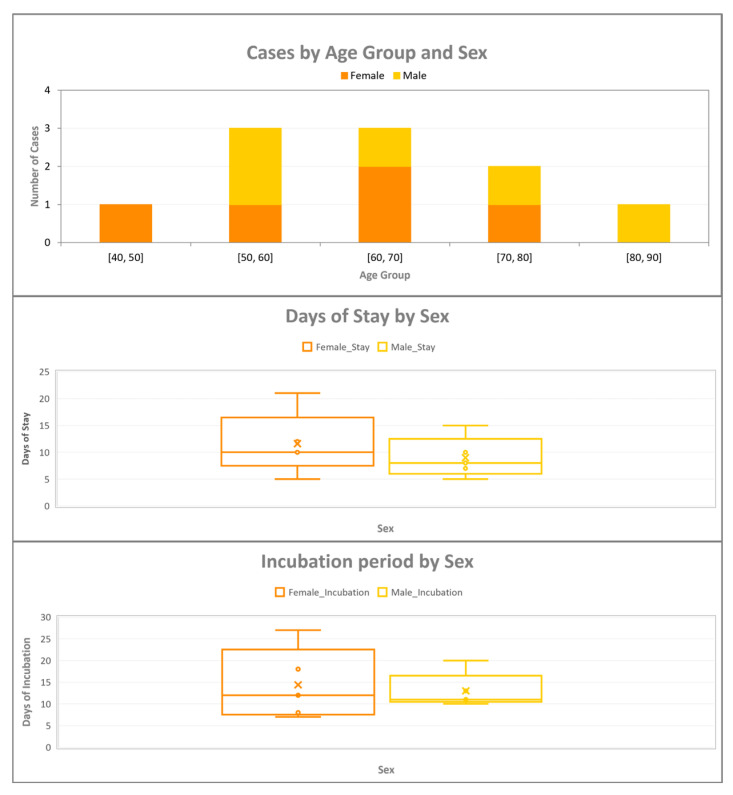
Distribution of Legionnaires’ disease cases according to demographic and epidemiological characteristics. The legend order has been revised to correspond to the color sequence of the stacked bars. The top panel illustrates the age–sex distribution of cases, the middle panel presents the duration of stay by sex, and the bottom panel depicts the incubation period by sex.
