# Supplementary material for: Beyond Microbiological Analysis: The Essential Role of Risk Assessment in Travel-Associated Legionnaires’ Disease Outbreak Investigations
Source: Pathogens. 2025 Oct 20;14(10):1059. doi: 10.3390/pathogens14101059 (PMC12567099; doi:10.3390/pathogens14101059)
Supplement: Supplementary file 1 [file pathogens-14-01059-s001.zip › pathogens-3907587-supplementary.pdf]

pathogens

MDPI

Supplementary Materials

Table S1. Check List According to the European Technical Guidelines 2017: minimizing the risk from *Legionella* infections in building water systems.

Item to check	Yes	No	Comment/Action required
**1. Assessment of the ability of premises personnel to control risk**			
Is there a person or persons appointed with responsibility for *Legionella* control?		no	
Is this person(s), and other concerned relevant staff, properly trained in the control of *Legionella* and able to demonstrate they understand the system(s), risk factors and control measures?		no	
If an external firm is providing help and advice, have efforts been taken to ensure the contractors and operatives on site are trained and competent in this task and there is adequate supervision?	yes		
**2. Assessment of the control measures (domestic cold and hot water temperatures and biocide levels)**			
Is there a continuous supply of source water of potable quality from a public utility?	yes		
Is there a private water supply used for source water (e.g. well, spring or bunkered water)?		no	
If there is a private supply, is there adequate point-of-entry treatment?		no	
Is there evidence (e.g. monitoring of appropriate sentinel points throughout the year) that the hot water temperatures of the entire hot water system are kept all times between 50°C and 60°C?		no	Temperature monitoring and recording began on 28/05/2025. Measurements are taken twice daily (morning and noon) in a different room each time, at the outlet and return points of the domestic hot water system. From 28/05/2025 to 10/06/2025, the temperature in the rooms ranged from 58°C to 62°C, the hot water outlet temperature ranged from 63°C to 66°C, and the return temperature ranged from 50°C to 52°C.

Is there evidence that the cold water temperatures of the entire cold water system are below 25°C?		no	
Are there other preventive methods in place (chlorine, chlorine dioxide, copper/silver ions, etc.)?	yes		Chlorine dioxide was used in 2025, while in previous years hydrogen peroxide was used.
Is there evidence that there is adequate monitoring and effective biocide levels maintained in the entire circuits and up to the outlets?		no	
**3. Assessment of other factors that may promote *Legionella* growth (low flow /stagnancy, scale, sediments, corrosion, etc.)**			
Is there evidence that all taps, showers and any other points of water use in all buildings are flushed for several minutes (sufficient to remove any stagnant water) on a weekly basis?		no	
Are showerheads, hoses and tap filters, aerators etc., TMVs and sieves cleaned, descaled and disinfected on a regular basis as advised in the risk assessment? (The frequency will depend on scale deposition and usage.)		no	
Is there pipework with intermittent or no water flow (bypasses, dead-legs, blind ends, areas not used or used intermittently, etc.) in any part of the water network?	yes		
Is there any visible or significant sediment, biofilm/slime, dirt, corrosion or scale deposit in any part of the water network?	yes		Some faucets show signs of rust and corrosion.
**4. Assessment of the cleaning and disinfecting practices**			
Are the calorifiers inspected, drained, cleaned and disinfected at least annually, and when a building is not used throughout the year, before the beginning of every season and after any maintenance?	yes		
Are cold water tanks inspected, cleaned and disinfected annually, and when a building is not used throughout the year before the beginning of every season?	yes		
Is the entire water network disinfected when a building is not used throughout the year before the beginning of every season?			

Are water filters (sand filters, multimedia filters, etc.) and softeners disinfected regularly, at least every three months? Are carbon filters (where used) replaced according to the manufacturer's instructions and before the beginning of each season?		There are no filters
Are there adequate written procedures for cleaning and disinfecting the water systems (e.g. 50 ppm chlorine for an hour)?	no	
**5. Assessment of the surveillance and monitoring practices and associated documents**		
Is there a written *Legionella* control programme in place for each system which could pose a risk?	no	
Is this programme suitable and sufficient for all the systems which could pose a risk of Legionnaires' disease in the premises?	no	
Are there regular records (logbook for example) of the critical monitoring activities kept on site (temperatures, chlorine levels, etc.)? Are appropriate actions taken to out-of-target results? Are these records reviewed by the responsible person(s)?	no	
Is a risk assessment review of the premises' water systems carried out regularly (at least every two years) or when there have been changes which may affect the risk assessment (e.g. change of frequency and type of use, key personnel)?	no	
Are both the risk assessment and operation of the control measures periodically audited by an independent and competent person?	no	
**6. Assessment of particular water systems (spa pools, wet cooling towers) present at the premises**		

If there is a spa pool, are there records to show that: • there are trained competent operatives on site? • there is continuous treatment with chlorine or bromine and pH adjustment (3-5 mg/1 chlorine or bromine and pH at 7.0-7.6)? • chlorine/bromine and pH is monitored at least three times/day? • the dosing system is checked on a daily basis to check pumps are working and the expected amount of chemicals has been used? • half the water is replaced each day? • sand filters are backwashed at the end of every day after the last person has left the pool? • the whole system, including the balance tank, is cleaned and disinfected once a week? • daily records are kept of the temperature and all water treatment readings (chlorine/bromine, pH)? • there has been an appropriate response to out-of-target results? • there is evidence to show they are checked regularly by responsible persons?	There are no spas
If there is an evaporative cooling tower, ensure that: • there is evidence that a trained and competent person has carried out a risk assessment; • there is a trained and competent person in charge of the cooling tower treatment and monitoring regime: • there is evidence that there is an adequate water treatment regime implemented (with effective biocides, corrosion inhibitors and adequate bleed-off rates as a minimum); • there is a daily check to ensure the dosing systems are working effectively and the expected volume of chemicals is being used; • entire cooling tower and associated pipes are cleaned and disinfected at least twice a year (and where a building is not used throughout the year and before the beginning of every season); • the system is inspected monthly for ensuring that drift eliminators are intact and firmly in place; • a microbiological monitoring system and a chemical one (e.g. chlorine or bromine treatment) is in place; • samples are taken to represent the worst-case scenario i.e., just before dosing (if intermittent) and at the furthest point from where the biocide enters the system.	There are no cooling towers
Check and list other at-risk water systems present at the building within the near vicinity with indication of their hygienic status, for example: • beach/outdoor pool showers • irrigation systems; • misting devices (e.g. in food display cabinets); • ornamental fountains/water features (indoor); • ornamental fountains/water features (outdoors); • humidifiers (air conditioning); • natural thermal springs; • vehicle washers; • solar systems; • grey water and rainwater recovery systems. Are there	There are:<br>outdoor pool showers<br>an irrigation system (drip type)<br>decorative fountains / water features (in outdoor areas)

any other systems in the vicinity which may pose a risk (e.g. wet scrubbers, industrial water systems, wastewater treatment plants/systems)? Others:

Table S2. Risk scores attributed to structural features of touristic-recreational facilities. The checklist has been adapted from the European Guidelines for Prevention and Control of Travel Associated Legionnaires' disease and from the National School of Public Health 2004 Athens Olympic Games Checklist for building water systems.

Legionella Prevention and Management Checklist			
	**Item to Check Yes /No**	**Points**	**Score**
	**General Testing Sites of the Water Network**		
1	The pressure on the meter is 1-12 atmospheres	-1	0
2	The filters are in good condition	-2	0
3	The sealing is in good condition	-2	0
4	Absence of leaks on the network	-2	0
5*	The storage tank is maintained in good condition and no sediments are observed inside it	-3	0
6	The water storage tanks have lids and wire mesh in each open air duct	-1	0
7	The amount of water stored is no greater than one day's use	-1	0
8*	The network is cleaned and disinfected when it is not under use for more than a month	-3	3
9*	The network and the tanks are cleaned with appropriate disinfectants at least once a year	-3	0
10	The water supply is not interrupted for long periods of time	-1	0
11	Unused taps are removed from the network	-2	1
12	Checking water network diagrams	-	
	**Cold Water Systems**		
13	Chillers are maintained in good condition	-1	
14	Chiller filters are maintained in good condition	-1	
	**Hot Water Systems**		
15	The system responds well at peak hours	-1	1
16	There is no change (increase or decrease) in water consumption	-1	1
17*	No standing water in piping for more than one week	-3	3
18 *	If No: Is a flushing procedure used?	-3	3
19*	Shower heads and taps are clean and free from scale	-3	0
	Water Heating and Storage Appliances		

20	The appliance is dried and checked	-1	0
21	The appliance is cleaned if necessary	-2	2
22	The hot water extraction duct is dried	-1	0
23	The appliance is maintained in accepted sanitary condition	-2	0
	**Faucets**		
24	Operated and maintained in accordance with the manufacturing instructions	-2	0
	**Fire Protection Water Supply**		
25	No backflow of water fire extinguishing system in the water supply network	-2	0
	**ANNEX I: Database Book**		
26	Are there regular records (logbook for example) of the critical monitoring activities kept on site (temperatures, chlorine levels, etc.)?	-2	2
27*	Random checks of water at least every 6 months	-3	3
28	In the control book (if any), there are no abnormal results	-2	3
29*	No *Legionella* detected the last six months (at a concentration of more than 1000 CFU/L)	-3	3
	**ANNEX II: Recordings**		
30*	Cold water is maintained at temperatures below 25 °C	-3	3
31	The cold-water temperature in taps is <25 °C after 2 min flushing	-2	3
32	The hot water temperature is >50 °C after 1 min flow	-2	3
33*	The difference in temperature between 2 successive measurements of hot water is NOT > 10 °C/1 min	-3	0
34	Hot water storage and circulation temp <60 °C	-2	2
35	There is NO thermal stratification of the water inside the heaters and storage water	-1	1
36	The water temperature is >60 °C when exiting the heating unit and >50 °C when returning to it	-2	0
37	Is the pH maintained at 7.2-7.8?	-2	0
38*	There is continuous treatment with chlorine at 0.2-0.5 mg/L?	-3	0
39	There is no taste and odor problem	-1	1
	Inspection results: (A) satisfactory result (0 to 7 points, <10% of the total negative score, no critical violation); (B) relatively satisfactory result (-8 to 14 points, 11-20% of the total negative score, or a critical violation); (C) unsatisfactory result (more than 14 points, >20% of the total negative score).		-38

Summary: Total negative score -38. Overall inspection classification: B – Relatively satisfactory (11–20% negative score, with ≥1 CCP failure). 8 CCP failures identified.

Table S3. Risk scores attributed to structural features of touristic-recreational facilities.

Item risk factors	Response	Risk score	Brief description	References	Score
Water Safety Plan	Implemented	0		[14,27,115]	0
	Not implemented	2			2
Opening or Closing period	No cases in open or closing periods	0		[14,27,115]	0
	Has cases in open or closing periods	2			2
Automated chlorination system	Automated chlorination system	0		[14,27,115]	0
	No automated chlorination system	2			2
Munithipal Population	<10.000	2		[14,27,115]	2
	> 10.000 residents	0			0
Facility type	Hotel, residence	2	*Legionella* is present more frequently in larger buildings because of the complexity of water system	[14,115]	2
	Guest houses, resorts, bed and breakfast	1			0
Activity period	Year-round	1	The seasonal use of the facilities promotes the proliferation of *Legionella* due to water stagnation	[14,27,115]	0
	Seasonal	2			2
Facility classification	0–1 star	1	The number of stars assigned to the facilities is directly proportional to the size of buildings and therefore, to the extension of the water network. This leads to greater variability in water temperatures and the formation of biofilms	[14,27,113,115]	0
	2–3 stars	2			2
	4–5 stars	3			0
Facility age	<10 years	1	Aged plumbing can promote the proliferation of *Legionella* due to biofilm and dead legs	[14]	0
	10–20 years	2			0
	>20 years	3			3
Time since last renovation	<10 years	1	Aged plumbing can promote the proliferation of *Legionella* due to biofilm and dead legs	[113]	0
	10–20 years	2			2
	>20 years	3			0
Number of floors	ground floor	1	The number of floors is directly proportional to the size of the buildings and, therefore, to the distribution of the water network on several levels. This can lead to variable distribution of the water temperature and the formation of biofilms	[14,27,115]	0
	2–5	2			2
	>5	3			0

Number of rooms per floor	<3<br>4–10<br>>10	1<br>2<br>3	Larger structures show greater contamination, because the hydraulic system is more complex and has a high number of water supply points	[14,27,115]	0<br>0<br>3
Number of rooms with showers	<10%<br>10%–40%<br>>40%	1<br>2<br>3	Aerosol from showers represents a potential exposure to *Legionella* if the pipes are colonized	[14,115]	0<br>0<br>3
Presence of wellness water services (whirlpool, sauna, swimming pool)	Yes<br>No	2<br>0	Recreational use of contaminated aerosolized water can be a source of legionellosis	[27,116]	2<br>0
	Maximum score	52		Total Score	30

Table S4. Risk scores attributed to water systems of touristic-recreational facilities.

Item risk factors	Response	Risk score	Brief description	References	
Source of water supply	Deep<br>Mixed<br>Superficial	1<br>2<br>3	The groundwater quality is preserved by soil filtration processes, compared to superficial source	[14]	0<br>2<br>0
Distribution network	With recirculation<br>Without recirculation	0<br>3	The absence of recirculation promotes biofilm, obstruction, stagnation of the water flow	[14]	0<br>0
Recirculation type	Total<br>Partial	1<br>2	The absence of a proper water recirculation system limits the treatment options adoptable in an emergency condition	[14]	1<br>0
Use of water softener	Yes<br>No	1<br>2	The hardness and calcium concentration are positively correlated to *Legionella*	[94]	1<br>0
Use of cold water storage tanks	No<br>Yes	1<br>2	The water storage or stagnation can encourage the proliferation of *Legionella*	[14]	0<br>2
Number of cold water storage tanks	1–3<br>4–8<br>>8	1<br>2<br>3	Excessive water storage or stagnation can encourage the proliferation of *Legionella*	[14]	1<br>0<br>0
Use of covers for the cold water storage tanks	Yes<br>No	1<br>2	The covers prevent the heating of the cold water which favors the proliferation of *Legionella*	[14]	1<br>0
Water temperature at the point of use	<20°– >50 °C<br>20°–50 °C	0<br>2	The optimal growth temperature for *Legionella* is 20°–50 °C	[14]	0<br>2
	Yes	1		[14]	0

Presence of treatment systems upstream of water heaters	No	2	Water heaters can be colonized by *Legionella* when the water temperature is optimal for its growth		2
Use of hot water storage tanks	No	1	*Legionella* probably concentrates on the bottom of the tank, where the temperature is a little lower	[14]	1
	Yes	2			0
Means of heating water	Storage tanks	2	Water stagnation favorable to the proliferation of *Legionella* may be present in the storage tanks	[14]	2
	Other	1			0
Use of good practices to prevent legionellosis	Yes	0	Good general hygiene practices and interventions to minimize exposure to specific risks	[14]	0
	No	5			0
Use of cleaning and sanitizing protocol	Yes	0	Cleaning and sanitizing of the water network reduce the risk factors for legionellosis are the foundation of all the prevention activities	[14]	0
	No	5			0
Periodic inspection, cleaning, and disinfection of storage tanks	Yes	0	The risk of *Legionella* contamination is foreseeable in the storage tanks of the water system if there are deposits that promote bacterial growth, such as rust, mud, scale and organic matter	[14]	0
	No	2			0
Frequency of inspection, cleaning, and disinfection of storage tanks	0–3 months	0	More frequent are the preventive actions, lowest is the risk of legionellosis	[14]	0
	4–6 months	1			0
	> months	2			2
Use of a register to record actions involving storage tanks	Yes	0	Compiling the register allows to track the interventions of control and prevention of legionellosis over time	[14]	0
	No	2			2
Use of a calendar of actions involving storage tanks	Yes	0	A timely and correct planning of actions decrease the risk of contracting legionellosis	[14]	2
	No	2			0
Use of a checklist of maintenance operations on storage tanks	Yes	0	A timely and correct planning of actions decrease the risk of contracting legionellosis	[14]	2
	No	2			0
Periodic replacement of shower heads and the jet fringes of water faucets	Yes	0	The biofilm on surfaces of hydraulic system can create a biological niche for *Legionella*	[14]	2
	No	2			0
Frequency of replacing shower heads and the jet fringes of water faucets	0–3 months	0	Increasing the frequency of management of water networks, the favorable conditions for the proliferation of *Legionella* would be avoided	[14]	0
	4–6 months	1			0
	>6 months	2			2
Microbiological monitoring of shower heads and the jet fringes of water faucets	Yes	0	Microbiological monitoring allows to control the livel of bacterial proliferation	[14]	0
	No	2			2
Inclusion of *Legionella* detection in microbiological monitoring	Yes	0	*Legionella* detection is one of the key elements to identify potential sources of infection	[14]	0
	No	1			1

Use of a register of actions involving shower heads and the jet fringes of water faucets	Yes	0	Compiling the register allows to track the interventions of control and prevention of legionellosis over time	[14]	2
	No	2			0
Use of a calendar of actions involving shower heads and the jet fringes of water faucets	Yes	0	A timely and correct planning of actions decrease the risk of contracting legionellosis	[14]	2
	No	2			0
Use of a checklist of maintenance operations involving shower heads and the jet fringes of water faucets	Yes	0	A timely and correct planning of actions decrease the risk of contracting legionellosis	[14]	2
	Maximum score	71		Total Score	36

Table S5. Physicochemical and microbiological parameters of selected water samples collected from the hotel's water distribution system, April–June 2025.

Date	Sample	Cl	pH	Temp	TVC@22° C cfu/ml	TVC@37°C cfu/ml	Coliform bacteria cfu/100ml	E. coli cfu/100ml	Intestinal enterococci cfu/100ml	*Ps. Aeruginosa* cfu/250ml	*Legionella spp.* ≥50 *CFU/L*
23/6/2025	Incoming Water from the Municipal Water Supply Network	0.3	7.3	24.8	123	108	0	0	0	0	no
23/6/2025	Outlet from the Hotel's Storage Tank	0.3	7.4	21.7			0	0	0	0	no
23/6/2025	Drinking Water from Tap Room	1.5	7.5	20.5			0	0	0	0	no
14/4/2025	Pool Water	1.8	7.5	20	0	0	0	0	0	0	no
23/5/2025	Drinking Water from Tap Room	1.8	7.5	25.9	45		0	0	0	0	no
22/6/2025	Boiler Room – Network Tap	1.5	7.5				39	0	0	0	yes
22/6/2025	Boiler Collector Return	1.5	7.5				61	0	0	0	no
22/6/2025	1st Boiler before Solar Heat Exchanger	1.5	7.5				8	0	0	0	no
22/6/2025	Water Tank Tap	1.5	7.5				32	0	0	0	no
22/6/2025	Boiler Outlet from Collector	1.5	7.5				39	0	0	0	no
22/6/2025	Kitchen – Vegetable Processing. Hand Washing Sink	1.5	7.5				17,000	0	0	0	yes
22/6/2025	Pool Bar Tap 2A	1.5	7.5				4	0	0	0	no
22/6/2025	Ice Machine Water	1.5	7.5				5	0	0	0	no
22/6/2025	Swimming Pool water	1.5	7.5				355	0	0	0	yes
22/6/2025	Swimming Pool Shower – Cold Direct	1.5	7.5				636	0	0	0	no
22/6/2025	Swimming Pool Shower – Cold Indirect	1.5	7.5				113	0	0	0	no
